# Meteorological Register

**Published:** 1814-02

**Authors:** C. Blunt

**Affiliations:** Philosophical Instrument Maker, No. 38, Tavistock-Street, Covent-Garden


### METEOROLOGICAL REGISTER.

### *From December* 25, 1813, *to January* 25, 1814.

Kept by C. BLUNT, Philosophical Instrument Maker, No. 38, Tavistock-Street, Covent-Garden.

| Moon. | Day. | Wind. | Barometrical Pressure. Max. | Min. | Temperature. Max. | Min. | |
|---|---|---|---|---|---|---|---|
| | 26 | NbE | 30·42 | 30·28 | 47 | 34 | Fair |
| | 27 | SW | 30·50 | 30·43 | 31 | 30 | Foggy |
| | 28 | NE | 30·39 | 30·31 | 30 | 28 | Foggy |
| | 29 | NE | 30·35 | 30·33 | 29 | 25 | Foggy |
| ◐ | 30 | NE | 30·36 | 30·30 | 32 | 23 | Foggy |
| | 31 | NE | 30·36 | 30·27 | 34 | 25 | Foggy |
| | 1 | NE | 30·19 | 30·08 | 36 | 24 | Foggy |
| | 2 | NE | 30·33 | 30·14 | 35 | 23 | Foggy |
| | 3 | WbN | 29·72 | 29·53 | 34 | 29 | Rain & Fog |
| | 4 | NE | 29·40 | 29·21 | 30 | 29 | Snow |
| | 5 | NE | 29·19 | 29·09 | 31 | 29 | Snow |
| ○ | 6 | NE | 29·47 | 29·16 | 30 | 25 | Frost |
| | 7 | N | 29·61 | 29·60 | 30 | 23 | Frost |
| | 8 | N | 29·60 | 29·52 | 30 | 19 | Frost |
| | 9 | N | 29·67 | 29·60 | 28 | 13* | Frost |
| | 10 | NE | 29·82 | 29·79 | 24 | 18 | Foggy |
| | 11 | NE | 29·84 | 29·64 | 24 | 20 | Clear |
| | 12 | N | 29·65 | 29·46 | 24 | 21 | Clear |
| ◑ | 13 | EbS | 29·84 | 29·76 | 25 | 21 | Clear |
| | 14 | EbS | 29·96 | 29·70 | 24 | 22 | Clear |
| | 15 | E | 29·58 | 29·50 | 26 | 23 | Clear |
| | 16 | E | 29·52 | 29·21 | 28 | 23 | Clear |
| | 17 | E | 29·69 | 29·27 | 30 | 22 | Snow |
| | 18 | NE | 29·10 | 29·06 | 29 | 20 | Snow |
| | 19 | EbN | 29·24 | 29·02 | 32 | 26 | Snow |
| | 20 | SEbE | 29·64 | 29·42 | 32 | 28 | Snow |
| ● | 21 | EbS | 29·76 | 29·75 | 28 | 20 | Snow |
| | 22 | E | 29·79 | 29·69 | 29 | 20 | Snow |
| | 23 | E | 29·75 | 29·73 | 30 | 19 | Clear |
| | 24 | E | 29·83 | 29·74 | 30 | 24 | Clear |
| | 25 | N | 29·88 | 29·82 | 31 | 24 | Clear |

Mean Height of the Barometer, 29·756.—Mean Temperature, 26·82.

* At Chelsea the thermometer sunk as low as 5°.

Dec. 26, 1813.—A fog, which continued to increase in density during the seven following days: the consequent darkness rendered it frequently necessary to burn lights within doors even in the middle of the day, and the extreme obscurity of the evenings, in most cases obliged the drivers of carriages to dismount and lead their horses, preceded by flambeaux. A common street lamp, with two wicks, disappeared at 40 feet distance.

Jan. 1, 1814.—During this day, the fog was at its greatest height; its effects were severely felt by invalids, even within the house. In the theatre at Drury-lane, when illuminated in the usual manner, a spectator in the lower boxes could not distinctly make out the persons of the company on the opposite side of the house; and the retired parts of the scenery on the stage were too indistinct to be seen with the naked eye. It is supposed this unusual fog arose, or was first noticed in the Downs; it thence followed the course of the Thames, spreading about eight or ten miles on its northern and southern banks. We have had no opportunity of ascertaining its westerly extension. No fog of so long duration is noticed in our registers since that of the year 1755, which lasted nine days, and preceded the great earthquake at Lisbon.

2d.—A slight thaw, during which the fog changed to a wet dew, and gradually disappeared; in the evening the frost set in again, and still continues (January 26). The fall of snow during this month is remarkable for its unusual quantity; the depth during the night of the 19th, exceeded 10 inches; in many parts of the country the drift is 12 feet in the open roads, which are consequently impassable to man or horse. The Thames is frozen over, and many persons have crossed it on the ice near the bridges of the metropolis; above Putney it has been passable since the 12th.

In consequence of the continued severity of the weather during the whole of the last month, *catarrhs* have been very prevalent, accompanied in many cases by an extreme debility, and frequently terminating in peripneumony. In some instances, where the active remedies of bleeding and blistering were required, they have proved fatal. *Scarlatina* has occasionally occurred in the metropolis and the vicinity, but, for the most part, in a mild form. *Measles* have also shewn themselves in particular situations; but, except in one case, where the patient was neglected till a late stage of the disorder, we have seen no instance of death from them; and, as far as our observations extend, we are decidedly of opinion, that there is no ground for the alarm which Dr. Watt has excited respecting their increased fatality.

